# EXERCISES AND NEUROMUSCULAR ELECTRIC STIMULATION FOR MEDIAL LONGITUDINAL ARCH: CLINICAL TRIAL

**DOI:** 10.1590/1413-785220233102e259598

**Published:** 2023-06-09

**Authors:** ANDRÉ SETTI PERSIANE, DAIANE MAGALHÃES GOMES NEGRÃO, RAONE DALTRO PARAGUASSU ALVES, DIEGO GALACE DE FREITAS, CLÁUDIO CAZARINI, VERA LÚCIA DOS SANTOS ALVES

**Affiliations:** 1. Faculdade de Ciências Médicas da Santa Casa de São Paulo, São Paulo, SP, Brazil.; 2. Centro Universitário Faculdade de Medicina do ABC (FMABC), Santo André, SP, Brazil.; 3. Irmandade da Santa Casa de Misericórdia de São Paulo, Department of Musculoskeletal Physical Therapy, São Paulo, SP, Brazil.

**Keywords:** Electrical stimulation therapy, Foot, Foot deformities, Talipes Valgus, Talipes Cavus, Terapia por Estimulação Elétrica, Pé, Deformidades do Pé, Pé Chato, Pé Cavo

## Abstract

**Objective:**

The extrinsic muscles, such as the posterior tibialis and long flexor of the hallux and the intrinsic of the foot, are part of the active subsystem of the central system of the foot and play an essential role in the control of the medial longitudinal arch resulting from difficulty in contracting the muscle, neuromuscular electrostimulation (NMES) becomes a resource combined with strengthening and recommended for rehabilitation. T this work aims to evaluate the effectiveness of NMES associated with exercise in deforming the medial longitudinal arch.

**Methods:**

This is a randomized blind clinical trial. 60 asymptomatic participants were divided into three groups: NMES, exercise and control. The NMES and exercise group performed seven exercises for the intrinsic and extrinsic muscles twice a week for 6 weeks, and the NMES group used an NMES associated with five exercises. Navicular height and medial longitudinal arch angle were taken before and after the intervention period.

**Results:**

No statistically significant differences existed between groups for navicular height and medial longitudinal arch angle.

**Conclusion:**

NMES associated with exercise does not change the characteristics of the medial longitudinal arch in association with asymptomatic. Level of Evidence I; Randomized clinical trial.

## RESUMO

**Objetivo:**

Os músculos extrínsecos, como o tibial posterior e flexor longo do hálux e os intrínsecos do pé fazem parte do subsistema ativo do foot core system e exercem papel essencial no controle do arco longitudinal medial. Devido à dificuldade na contração desses músculos, a eletroestimulação neuromuscular (EENM) torna-se um recurso aliado ao fortalecimento e é recomendada para reabilitação. O objetivo desse trabalho é avaliar a eficácia da EENM associada ao exercício na deformação do arco longitudinal medial.

**Métodos:**

Este é um ensaio clínico randomizado cego. 60 participantes assintomáticos foram divididos em três grupos: EENM, exercício e controle. O grupo EENM e exercício realizaram sete exercícios para os músculos intrínsecos e extrínsecos duas vezes por semana por seis semanas, sendo o grupo EENM utilizou a EENM associada a cinco exercícios. A altura do navicular e o ângulo do arco longitudinal medial foram medidos antes e após o período de intervenção.

**Resultados:**

Não houve diferenças estatisticamente significativas entre os grupos para a altura do navicular e ângulo do arco longitudinal medial.

**Conclusão:**

A EENM associada ao exercício não altera as características do arco longitudinal medial em indivíduos assintomáticos. Nível de Evidência I; Estudo Clínico Randomizado.

## INTRODUCTION

The main structure of load bearing and shock absorption of the foot is the medial longitudinal arch. Changes in medial longitudinal arch can affect the foot biomechanics, change the distribution of plantar loads in individuals with injuries in their feet or in any other joints, and cause pain.^1 - 3^

The foot core system is a paradigm for understanding the medial longitudinal arch functionality that compares it to the spine stability. There are three subsystems in this theory: the passive, including the foot bones, plantar fascia and ligaments; the neural, with muscle and tendinous receptors, local and global, in ligaments and on the plantar skin; and the active, with intrinsic muscles, local and extrinsic stabilizers, that are essential for the foot global movements.^4^

Several types of exercise have been proposed to increase the muscle activation with a focus on the active contribution of medial longitudinal arch. However, these are muscles difficult to feel and contract.^5 , 6^ If muscles regulate the deformity and stiffness of the medial longitudinal arch, there would be a possibility that the electric stimulation applied to intrinsic muscles could affect the natural contraction ability, resulting in increasing of the height and decreasing of the medial longitudinal arch length.^7^

The idea of stimulating the medial longitudinal arch with neuromuscular electric stimulation (NMES) as a way to activate these muscles seems reasonable and logical. Our objective was thus to evaluate the effect of NMES and of NMES plus exercising on anatomical changes of the medial longitudinal arch.

## METHODS

### Trial design

This parallel randomized controlled trial involved sedentary adults (not practicing any physical activity) without foot pain who were evaluated in a physiotherapy service of a university hospital between January 2017 and March 2018. The protocol was approved by the institutional review board (62766716.4.0000.5479) and registered at clinicaltrials.gov (NCT03117244). All participants signed informed consent forms. We report this trial according to the CONSORT Statement of Randomized Trials, especially the extension for Nonpharmacologic treatments^8^ and TIDieR reporting guideline.^9^

### Participants

We recruited participants for this trial through the institutional and the researchers’ personal social medial channels. We invited them to come to our physiotherapy clinic for the initial screening, which included personal health history and demographics and basic anthropometry and physical examination, as well as exercising habits. We excluded individuals reporting neurological diseases, and any foot or leg fracture, muscular or joint injury or surgery in the previous 12 months. We also excluded participants with previous or current rigid flat foot or valgus calcaneus higher than 10 degrees.

#### Interventions and groups

We allocated participants into three comparison groups: the Exercise, the NMES and the Control groups. In the Exercise group, participants received individual training twice a week for six weeks, at each participant’s most convenient time (morning or afternoon). In the NMES, they received the same exercises plus electric stimulation as described below, twice a week for six weeks, also at the most convenient time for the individual. Participants randomized to the control group were examined and then told to keep their routines and activities of daily life. We just asked them to come back to the service in six weeks for a new evaluation.

**Figure 1:**
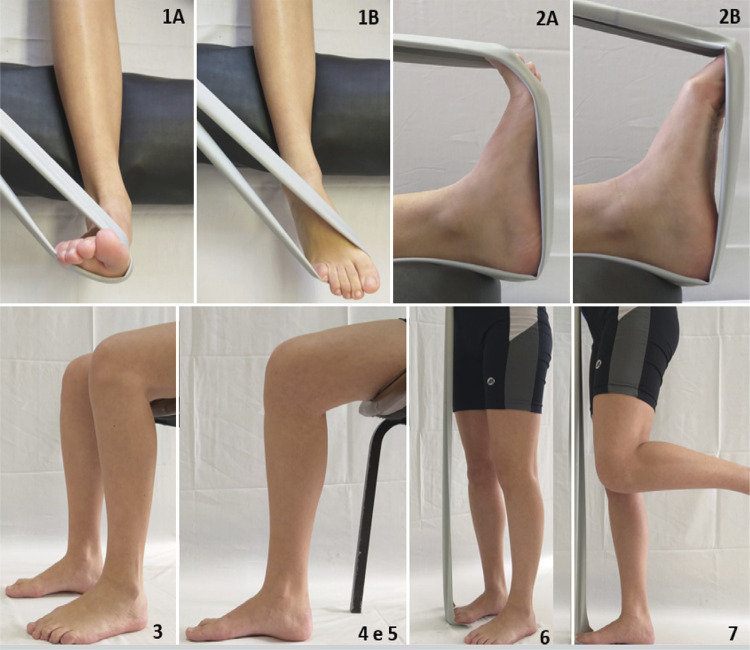
Stances for the proposed exercises for intrinsic and extrinsic muscles of the foot. 1: initial (A) and final (B) stances for posterior tibialis muscle exercise; 2: initial (A) and final (B) stances for the long flexor of the hallux exercise; 3: stance for the short-foot exercise with bipedal support while sitting; 4 and 5: stance for the short-foot exercise with unipedal support while sitting (picture showing the support on the right foot); 6: stance for exercise for intrinsic and extrinsic muscle while standing and using a rubber band; 7: stance for the single-leg exercise for intrinsic and extrinsic muscle with unipedal support.

The participants in the Exercise group performed a total of seven movements as described in Figure 1 and Table 1 .

**Table 1:** Intrinsic and extrinsic foot muscles exercise protocol for the intervention groups.

Muscle	Position	Frequency per week					
		1st	2nd	3rd	4th	5th	6th
Tibial posterior	Lying	3x15 r	3x15 r	3x15 r	3x15 r	3x30 r	3x30 r
Long flexor of the hallux	Lying	3x15 r	3x15 r	3x15 r	3x15 r	3x30 r	3x30 r
Intrinsic	Sitting-bipedal	3x15 r	3x15 r	3x15 r	3x15 r	3x30 r	3x30 r
Intrinsic	Sitting-unipedal	3x15 r	3x15 r	3x15 r	3x15 r	3x30 r	3x30 r
Intrinsic	Sitting-unipedal	3x30 s	3x30 s	3x30 s	3x30 s	3x60 s	3x60 s
Intrinsic and extrinsic	Standing-bipedal	3x30 s	3x30 s	3x30 s	3x30 s	3x60 s	3x60 s
Intrinsic and extrinsic	Standing-unipedal	3x30 s	3x30 s	3x30 s	3x30 s	3x60 s	3x60 s

r=repetitions; s=seconds; #=Stance shown in the Figure 1 panel.

Figure 1 shows the stance for each exercise and the muscles activated in the movements. The same exercises (intensity and duration) were proposed for all participants in this group, with no modifications according to anthropometry.

In the NMES group, during the exercises numbered 1 to 5 (as shown in Figure 1 ), participants also received electrical stimulation to the foot. We applied the depolarized, biphasic, symmetrical current with rectangular pulses of medium frequency modulated in low using a pulse generator (Sonophasys, EUS.0503, KLD Biosistemas, São Paulo, Brasil), and two self-adhesive silicone electrodes (Self-Adhesive Electrode Valutrode 5x5cm, Arktus, Santa Tereza do Oeste, Paraná, Brazil) placed in the region of the muscular belly of the flexor halluci, posterior tibialis and muscles intrinsic of the foot. The carrier frequency was 2500Hz, the modulation frequency was 50Hz, with an output duty cycle of 20%, one second up and down ramp and an on-and-off time with a 1:1 ratio, with the on proportional to the expenditure to perform the series of exercises. The same physiotherapist administered the interventions (the exercises and neuromuscular electric stimulation) for all participants in both groups.

## Evaluations and outcomes

For two weeks, we trained an independent physical therapist (author RDPA), with five years of experience, to perform the evaluations for this study. In the training we focused on anatomical structures palpation, identification of reference points and the measurements to be taken.

After training, the physical therapist evaluated 10 healthy volunteers as a pilot study, in two occasions with a one-week interval, and we registered these measurements. We calculated the intraclass correlation coefficient between the two measurements of the same individual, presetting the rule that a coefficient lower than 0.4 would not be acceptable.^10^

The evaluator took the basic demographic and clinical history of the included participants. Then, he measured the angle of the calcaneus, with the patient lying in prone position, with feet off the gurney. He palpated the calcaneus medially and laterally and bisected it, marking its lower and middle points, to form a line between the points. This way, he identified the subtalar neutral. With palpation of the talus, he measured the varus or valgus of the calcaneus using a plastic goniometer with protractor and two 20cm rulers (SH5205, Carci, São Paulo, Brasil).^11^

The therapist then asked the participant to sit, with hips, knees and ankles flexed at 90 degrees, and identified other anatomical points with a marker: the center of the medial malleolus, the tuberosity of the navicular and the head of the first metatarsus. Next, he palpated the lateral and medial aspects of the talus, with the subtalar joint in neutral position and measured the medial longitudinal arch angle and the navicular height. The therapist repeated these measurements with the participant standing with bipedal support, with the subtalar in a relaxed position.^11^

To measure the medial longitudinal arch angle, the evaluator placed the center of the goniometer in the tuberosity of the navicular, with its ends facing the center of the medial malleolus and the head of the first metatarsus.^12^ For the navicular height, he measured the distance (in centimeters) between the ground and the tuberosity of the navicular.^12^ All measurements were made in both feet of each participant by same evaluator.

## Randomization and blinding

The author DMGN performed the randomization for this study using a list from the randomization.com (website). We generated a randomization sequence for 60 participants initially using the first and original generator that uses the method of randomly permuted blocks. When the participant arrived for the preliminary evaluation for inclusion, if the individual was considered eligible and consented to participate, DMGN consulted the list and warned the physiotherapist about the allocation.

In this trial, due to the nature of the interventions used, it was not possible to blind participants: they all knew what intervention they were receiving or not. The physical therapist who administered the interventions, guiding the exercises and applying the electric stimulation. We asked participants to hide the allocation from this evaluator (i.e., not telling him if they performed exercises or not, for example).

## Sample size and statistical analysis

We calculated sample size (ANOVA) and data from the pilot study (during the evaluator training). We adopted a significance level of 5%, power of 80% and the navicular height as the primary outcome, considering as significant a minimum of 20% of difference between means, with a standard deviation of 0.75. According to these assumptions, the sample size should be of 16 participants per group. Assuming some loss, we worked with a sample size of 20 participants per group.

For the intraclass correlation coefficient (ICC) calculation, for the pilot study, we determined the standard error measurement with standard deviation between the first and the second measurements, with the standard deviation multiplied by the square root (1 – ICC).^13^

We compared the study evaluations between groups and between moments (before and after the intervention). For this, we used the Shapiro-Wilk normality test to verify distribution. We described the measurements using medians, minimum and maximum values and used the Kruskal Wallis test for the non-parametric observations. For the parametric observations, we used means, standard deviations and the ANOVA test. The level of significance adopted for all tests in this study was 5% and the software was SPSS version 13.1.

## RESULTS

**Figure 2:**
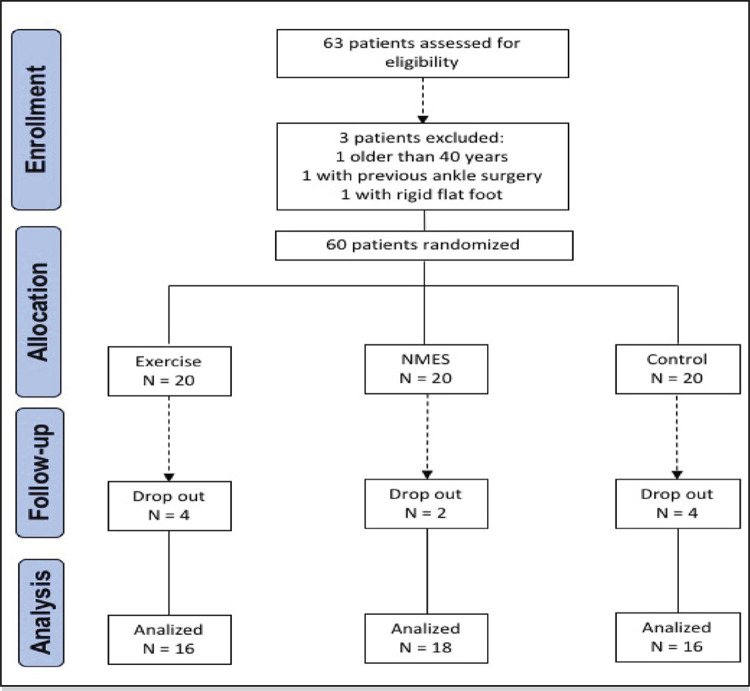
Flowchart of patients’ inclusion and exclusion in the study.

In the study period, we recruited 60 participants, and 50 of these completed the follow-up, as shown in Figure 2 . The reason for dropouts in the intervention groups was schedule conflicts with work or personal appointments.

Table 2 shows anthropometric evaluations and the similarity between groups at baseline.

**Table 2:** Baseline demographic and anthropometric data per group (n=48).

	Groups			
Variable	Exercise(n=16) Mean(SD)	NMES(n=18) Mean(SD)	Control(n=16) Mean(SD)	p
Age (years)	26(5)	27(5)	26(5)	0.519
Sex (Female/Male)	11/5	14/4	12/4	-----
Height (m)	1.65(0.1)	1.65(0.1)	1.65(0.1)	0.930
Weight (kg)	60.8(9.9)	69.3(18.8)	65.2(13.4)	0.367
BMI (kg/m^2^)	22.1(2.2)	25.1(5.2)	23.7(3.7)	0.171

NMES=neuromuscular electric stimulation; SD=standard deviation; BMI=body mass index.

For the pilot evaluation, the ICC and the SEM between measurements were 0.98 cm and 0.15 degrees for navicular height and medial longitudinal arch angle respectively in the neutral position of the subtalar, and 0.98 cm and 0.11 degrees for the relaxed position, as well as 0.97 and 0.02 cm for neutral position of the navicular height, 0.92 and 0.06 cm for the navicular height for the relaxed position. This means the variation was acceptable.

The medial longitudinal arch angle and navicular height measurements (respectively on Tables 3 and 4 ) show that neither exercise nor electric stimulation resulted in significant outcome changes.

**Table 3:** Mean, minimum and maximum medial longitudinal arch angle measurements per groups.

Variable	Exercise(n=16)	NMES(n=18)	Control(n=16)	p
SIT pre right	150 (144-155)	146 (142-150)	146 (140-151)	0.439
BIP pre right	149 (144-153)	144 (140-148)	146 (141-150)	0.318
SIT pos right	151 (147-155)	148 (146-151)	149 (146-153)	0.555
BIP pos right*	148 (138-159)	146 (138-158)	150 (136-155)	0.344
SIT pre left	151 (143-158)	148 (145-151)	150 (145-155)	0.730
BIP pre left	149 (142-155)	144 (141-147)	147 (142-152)	0.336
SIT pos left	150 (146-154)	148 (147-150)	151 (145-156)	0.626
BIP pos left	149 (145-153)	144 (141-147)	147 (143-151)	0.186

*Median value. NMES=neuromuscular electric stimulation; SIT=sitting stance; BIP=bipedal support.

**Table 4:** Mean and minimum and maximum navicular height measurements per group (cm).

Variable	Exercise(n=16)	NMES(n=18)	Control(n=16)	p
SIT pre right	5.0 (4.6-5.3)	5.0 (4.7-5.3)	5.2 (4.8-5.6)	0.520
BIP pre right	4.3 (4.0-4.7)	4.1 (3.8-4.4)	4.5 (4.0-5.0)	0.295
SIT pos right	5.3 (3.8-6.0)	5.0 (4.3-6.2)	5.5 (4.3-6.4)	0.595
BIP pos right*	4.4 (4.1-4.8)	4.3 (4.0-4.6)	4.7 (4.3-5.1)	0.236
SIT pre left	5.0 (4.6-5.5)	4.8 (4.5-5.1)	5.2 (4.8-5.6)	0.285
BIP pre left	4.3 (4.1-4.7)	4.0 (3.8-4.3)	4.5 (4.0-5.1)	0.146
SIT pos left	5.1 (4.8-5.5)	4.9 (4.6-5.3)	5.3 (4.8-5.7)	0.408
BIP pos left	4.5 (4.2-4.8)	4.3 (3.9-4.6)	4.7 (4.2-5.1)	0.260

*Median value. NMES=neuromuscular electric stimulation; SIT=sitting stance; BIP=bipedal support.

## DISCUSSION

In this randomized controlled trial, exercising only or with electric stimulation did not result in any difference in the medial longitudinal arch measurements. To our knowledge, this is the first randomized trial using NMES and exercises assessing the changes in the medial longitudinal arch.

Typical values for navicular height were between 3.6 and 5.5 cm and 130 and 152 degrees for medial longitudinal arch angle in a study in Denmark.^12^ Our participants had values within these ranges both before and after exercising and electric stimulation, indicating that, if any, the effects of the intervention were not evidenced by anatomical changes.

Short-foot exercises can reactivate muscular components of the core system that may be inactive, allowing these muscles to contribute to the absorption and propulsion during activities involving the foot,^6^ such as walking and standing. Mulligan et al observed improvements of the medial longitudinal arch and the dynamic balance of the foot after four weeks of intrinsic muscle at-home training.^14^ Hashimoto et al also evaluated the effects of strength training for the intrinsic flexor muscles. The authors measured the medial longitudinal arch length and transverse arch of the foot, after an eight-week program with 200 repetitions a day, three times a week, with a load of three kilos. They observed increased strength and decreased length of the arches.^15^ However, both were before-and-after studies, with no control group.^14 , 15^

The motivation for this study was the lack of properly conducted randomized clinical trials evaluating the value of adding electrical stimulation to exercise in the rehabilitation or freeing of the core foot.^4 , 6^ Kelly et al thought about the possibility of using a direct current to stimulate the hallux abductor, short finger flexor and plantar square. The authors observed transient changes in navicular height and medial longitudinal arch angle through 3D kinematics, which probably fired the intrinsic muscles to control stiffness and deformation of the medial longitudinal arch. The experiment, however, was small, with nine healthy males, and with no control group.^7^

Recently, Ebrecht et al^16^ conducted a randomized trial on the effect of an NMES intervention on intrinsic foot muscles cross-sectional area as a proxy for muscle strength. The authors aimed to verify if NMES would change the cross-sectional area as measured by ultrasound, improve arch stability and reduce muscle fatigue. The measurements were made after 20 minutes of running in a treadmill, barefoot, for all participants (except the passive control group), with subgroup analysis for experienced or beginner runners. Arch stability and fatigue were evaluated through the static navicular drop. No strengthening effect was verified of the intrinsic foot muscles using NMES. However, there was little information on the NMES parameters of application and the authors themselves questioned if the intervention had been too short or the cross-sectional area and the navicular drop would be suitable to display muscle strength. The small sample size, especially for subgroup analyses, is a concern too. The authors suggested that a study with people who do not exercise was needed.

Using NMES in healthy muscles is a controversial issue in the literature, but studies have investigated adding NMES with exercise for muscles of the leg, some with positive results,^17^ others without.^18^ One explanation for the failure of NMES in these studies would be that in general they used participants with no neural or mechanical impairments whereas in a physical rehabilitation context of injured muscles or wasting or denervation following periods or immobilization maybe it could have detectable effects.^19^ We opted, thus, to choose a simple and basic measurement, possible to be performed without special equipment, and an area of the body not explored by well-conducted and reported RCTs, the medial longitudinal arch of the foot.

A limitation of our study would be that we did not classify the different types of feet (normal, pronated and supine) at baseline. However, we do not have data on the prevalence of foot pronation in our population, and the only reference data for “normality” available are based on populations that differ substantially in ethnicity and anthropometry^12^ from ours.

The participants in our study intervention groups trained twice a week, but the literature is controversial as to the ideal frequency of exercises to gain muscle strength. A recent systematic review with meta-analysis with subgroup analysis found that the training frequency produced better results for multiarticular exercises, training of upper limbs, for young adults and for the female sex. No significant association was found between the frequency and the strength gain for uniarticular exercises and training of the lower limbs for a male, middle-aged and elderly population.^20^ Again, this shows that the disparity of training protocols, and not the NMES per se, could be responsible for the lack of effects we found. We studied young adults, with 74% of females, twice a week, but there is no evidence that an increase in exercise frequency would help.

Future studies should focus on the motor control of the muscles involved, that is, they must be active at the moment of the support and impulse phase of walking and running and interventions must be focused on this. The medial longitudinal arch should be the focus of investigations, including static and dynamic deformation. The strengthening of medial longitudinal arch muscles should be studied in symptomatic patients for foot and ankle disorders. However, the outcomes of the work must be better designed, analyzed and reported by researchers, allowing the comparison between protocols.

## CONCLUSION

NMES associated with exercise does not change the characteristics of the medial longitudinal arch in association with asymptomatic.
